# Intestinal Ultrasonography as a Tool for Monitoring Disease Activity in Patients with Ulcerative Colitis

**DOI:** 10.1155/2022/3339866

**Published:** 2022-07-09

**Authors:** Milica Stojkovic Lalosevic, Aleksandra Sokic Milutinovic, Vera Matovic Zaric, Iva Lolic, Aleksandar Toplicanin, Sanja Dragasevic, Mirjana Stojkovic, Marija Stojanovic, Marko Aleksic, Mihailo Stjepanovic, Jelena Martinov Nestorov, Dusan Dj. Popovic, Tijana Glisic

**Affiliations:** ^1^Clinic of Gastroenterology and Hepatology, Clinical Center of Serbia, Belgrade, Serbia; ^2^Faculty of Medicine, University of Belgrade, Belgrade, Serbia

## Abstract

**Background:**

Ultrasonography is a noninvasive, inexpensive, and widely available diagnostic tool. In the last two decades, the development of ultrasound techniques and equipment has significantly increased the usage of intestine ultrasound (US) in the assessment of the gastrointestinal tract in patients with inflammatory bowel disease (IBD). Although current guidelines suggest routine utilization of US in patients with Crohn's disease, data regarding US usage in ulcerative colitis are still scarce. We aimed to assess the reliability of intestinal ultrasonography in the assessment of disease activity and extension of patients with ulcerative colitis.

**Methods:**

Fifty-five patients with a histologically confirmed diagnosis of ulcerative colitis, treated at University Clinical Center of Serbia in the period from 2019 to 2022 were included in this retrospective observational study. The data were obtained from the patient's medical records including history, laboratory, US, and endoscopy findings. US examined parameters were as following: bowel wall thickness (BWT), presence of fat wrapping, wall layer stratification, mesenteric hypertrophy, presence of enlarged mesenteric lymph nodes, and absence or presence of ascites.

**Results:**

Our results suggest that there is a strong correlation of BWT and colonoscopy findings regarding disease extension (*r* = 0.524, *p*=0.01, *p* < 0.05). Furthermore, our results have shown a statistically significant correlation of BWT with the Mayo endoscopic score (*r* = 0.434, *p*=0.01, *p* < 0.05), disease activity score (*r* = 0.369,*p*=0.01, *p* < 0.05), degree of ulcerative colitis burden of luminal inflammation (*r* = 0.366, *p*=0.01, *p* < 0.05), and Geboes index (*r* = 0.298, *p*=0.027, *p* < 0.05). Overall accuracy of US for disease extension and activity was statistically significant (*p* < 0.05).

**Conclusions:**

Our results suggest that US is a moderately accurate method for the assessment of disease activity and localization in patients with UC.

## 1. Introduction

Inflammatory bowel diseases (IBD) are chronic inflammatory conditions of unknown etiology. Episodes of inflammation's remission and relapses in various parts of the gastrointestinal (GI) tract are characteristic of IBD [1, 2]. Three different subtypes of IBD are recognized: ulcerative colitis, Crohn's disease, and indeterminate colitis. Ulcerative colitis (UC) and Crohn's disease (CD) differ in pathogenesis, localization, endoscopic, and histopathological findings [3]. The term indeterminate colitis is used in cases of inability to distinguish UC from CD based on endoscopic and histopathological features [4]. In UC, mucosal or submucosal inflammation is limited to the rectum and colon, while in CD transmural inflammation can affect any segment of the GI tract [5]. Until recently, clinical remission or absence of disease symptoms was considered a therapeutic success in IBD. However, a better understanding of the complex pathophysiological mechanisms of inflammation has set a new goal in the treatment of IBD. Achieving endoscopic as well as histological healing should provide better disease control and serve as an indicator of therapeutic response. To achieve stable remission, frequent monitoring of these patients is necessary.

Colonoscopy with histopathological analysis is the gold standard in diagnosing and follow-up of IBD patients. However, it is an invasive diagnostic procedure, generally not well tolerated, due to the required bowel preparation and discomfort or pain during the procedure [6].

In the last two decades, the development of ultrasound (US) techniques and equipment has significantly increased the role of intestinal US in the assessment of GI tract in patients with IBD. US is a noninvasive, inexpensive, widely available diagnostic tool, well accepted, and tolerated by the patients. Intestinal US was first applied in CD for the assessment of transmural inflammation, and nowadays, is an instrument for assessing disease activity, complications, and therapy response [7–9]. The clinical utility of intestinal US in patients with UC is not as well established as in CD [8, 10]. International guidelines recognize US in the diagnostic algorithm of IBD patients; however, there is a lack of standardization and general agreement on specific intestinal US parameters [11, 12]. Recently, an expert panel assessed the efficacy of US in UC to identify reliable parameters for diagnosis establishment, as well as disease monitoring. Among others, BWT, parietal blood flow, Doppler signal, wall layer stratification, and fatty wrapping showed promising results and should further be evaluated [13].

Our retrospective study aimed to assess the reliability of intestinal US in the evaluation of the disease extent and activity in patients with UC.

## 2. Material and Methods

This retrospective study was conducted from November 2019 to January 2022 at the Emergency Department, Clinic of Gastroenterology and Hepatology, University Clinical Center of Serbia.

Criteria for inclusion in the study were developed by following internationally accepted endoscopic, radiological, and histological standards for the diagnosis of IBD in patients over 18 years of age [14, 15]. Criteria for exclusion from the study were patients under 18 years of age, patients with proctitis, histologically proved indeterminate colitis, diagnosis of malignant tumor, and acute complications such as severe bleeding or toxic megacolon.

### 2.1. Data Collection

We collected the following data from electronic medical records: age, gender, localization of the disease, and time of diagnosis establishment. Blood samples were collected and analyzed for hemoglobin (Hb), white blood cells (WBC), platelets (PLT), sedimentation (SE), C-reactive protein (CRP), procalcitonin (pct), albumin (Alb), D-dimer, serum iron (sFe), ferritin, and stool samples were collected and analyzed for fecal calprotectin (FCP). Fecal calprotectin was measured using a validated enzyme-linked immunosorbent assay (ELISA). The upper limit of detection of the FCP test was 50 *μ*g/g.

### 2.2. Colonoscopy

Total colonoscopy with limited insufflation of air (in severe UC patients, to minimize the risk of acute traumatic dilation or perforation of the colon) was performed in all patients for diagnostic purposes as well as for the assessment of disease severity in 72 h after admission. Colonoscopy findings were scored according to the Mayo endoscopic score (MES) for each segment: 0 = normal or inactive disease; 1 = mild (erythema, decreased vascular pattern, mild friability); 2 = moderate (marked erythema, absent vascular pattern, friability, erosions); 3 = severe (spontaneous bleeding, ulceration) [16]. The colon involvement was defined as proctitis, left-side colitis, and pancolitis. The extent of disease was scored according to the Montreal classification: E1 = involvement limited to the rectum (that is, the proximal extent of inflammation is distal to the rectosigmoid junction); E2 = involvement limited to a proportion of the colorectum distal to the splenic flexure; E3 = involvement extends proximally to the splenic flexure [17]. Additionally, we calculated the Degree of Ulcerative colitis, Burden by Luminal Inflammation score (DUBLIN score) as a result of MES and disease extent [18]. Furthermore, the disease activity index was calculated (DAI) [19].

### 2.3. Intestinal Ultrasonography

All patients underwent transabdominal ultrasonography after at least 5 h of fasting, on the first day of admission. In all patients, intestinal US was performed at least 24 h prior to endoscopy by experienced sonographers (at least 10 years of experience) with a Samsung Medison ultrasound device using CA2-8AD convex transducer (frequency 2.0–8.0 MHz) and LA3-16AD linear transducer (frequency 3.0–16.0 MHz). Patients generally remained supine during the examinations or were moved to the decubitus position as needed. First, a convex transducer was used, followed by a high-frequency linear-array transducer for detailed evaluation. The following intestine US parameters were recorded during the procedure: bowel wall thickness (BWT), presence of fat wrapping (hyperechoic fat around the bowel), wall layer stratification (WLS), mesenteric hypertrophy, presence of enlarged mesenteric lymph nodes, and absence or presence of ascites. BWT was measured in each patient in the region of the sigmoid colon.

### 2.4. Ethical Consideration

The study was in accordance with the regulations of the ethics committee of our institution (638/22). The study was conducted according to the principles of the Helsinki Declaration (1989).

### 2.5. Statistical Analysis

Statistical analysis was performed with SPSS ver. 20.0 (IBM, Chicago, IL, USA) (Student's *t*-test, Mann–Whitney test, chi-square test). Demographic and clinical characteristics were summarized by basic descriptive statistics, including means, medians, interquartile range (IQR), standard deviations, and percentages. The Kolmogorov–Smirnov test was used for examining the normality of distribution. The ANOVA test was used for examination of the difference between groups for normally distributed variables. The correlation was examined using Pearson's and Spearman's correlation tests. Measure of the Agreement-Kappa test defined as a measuring tool for inter-rater reliability was used for examination of agreement of intestinal US with colonoscopy. Categories of interpretation of Kappa results, advised by Cohen, were as follows: values < 0 suggesting no agreement, 0.01–0.20 none to slight agreement, 0.21–0.40 fair, 0.41–0.60 moderate, 0.61–0.80 strong, and 0.81–1 almost perfect agreement [20]. Cutoff values, with sensitivity and specificity, were calculated in accordance with receiver operator characteristic (ROC) analysis, with an additional calculation of the area under the ROC (AUROC). The Youden index was used to determine the best cutoff values. Logistic regression analysis was used for the calculation of odds ratios (ORs) and 95% confidence intervals (CIs). Results were considered statistically significant for a *p* values less than 0.05.

## 3. Results

Baseline characteristics of our cohort were presented in Table 1. Our study included 55 patients with UC. Among the patients included in this study, 33 were male gender (60%), while 22 were female gender (40%), with mean age of 44.2 years. The median value of the DAI score was 9.36 (range 0–12). The average disease duration prior to inclusion in this study was 6.69 years.

### 3.1. Colonoscopy Findings

In the majority of the patients (64%), the disease was found proximal to the splenic flexure (E3), while the rest of the patients had left-side (E2) colitis (36%) (Table 1). MES in 39 patients (71%) suggested severe disease, in 10 patients (18%) moderate, in 5 patients (9%) mild disease, while in 1 patient (2%) the disease was inactive. The mean DUBLIN score in our cohort of patients was 6.33.

### 3.2. Histologic Activity

Disease activity was additionally confirmed through histopathological findings. Our results suggest a mean Geboes score of 4.4 (ranging from 1.1 to 5.4).

### 3.3. Ultrasonography Parameters

The mean BWT was 5.54 ± 0.32 mm when our patients were admitted to the hospital. According to ultrasonography findings, ulcerative colitis was extended to E3 in 55% of the patients, and E2 in 45% of patients. The fat wrapping was present in 43 patients (78%) and wall layer stratification was seen in 46 patients (84%). Mesenteric hypertrophy was present in 14 patients (26%), while 41 patients (75%) showed no significant mesenteric hypertrophy. Enlarged lymph glands were seen in 12 patients (22%), and free fluid surrounding the affected part of the colon was seen in 9 patients (16%).

### 3.4. Correlation of Disease Extension Examined by Ultrasonography and Colonoscopy

We analyzed the correlation of disease extension examined by ultrasonography and colonoscopy, and our results suggest that there is a strong correlation of these two methods (*r* = 0.524, *p*=0.01, *p* < 0.05). The overall accuracy of ultrasonography for disease extension was statistically significant with moderate straight of agreement (*κ* = 0.515, SE (k) = 0.115, *p* < 0.05). Sensitivity of ultrasonography in the assessment of disease localization was 74.3%, while specificity was 80%.

### 3.5. Correlation of Disease Activity Examined by Ultrasonography and Colonoscopy

We analyzed correlation of markers of disease activity with ultrasonographic parameters and found statistically significant correlation of BWT with MES (*r* = 0.434, *p*=0.01, *p* < 0.055), DAI score (*r* = 0.369, *p*=0.01, *p* < 0.05), DUBLIN (*r* = 0.366, *p*=0.01, *p* < 0.05), and Geboes index (*r* = 0.298,*p*=0.027, *p* < 0.05). Additionally, there was a significant correlation between fat wrapping, wall layer stratification and MES, DAI, DUBLIN, and Geboes index (*p* < 0.05). There was no statistically significant correlation of mesenteric hypertrophy, presence of enlarged mesenteric lymph nodes, and absence or presence of ascites with BWT, MES, DAI, DUBLIN, and Geboes index (*p* > 0.05).

Additionally, our results showed a statistically significant difference in BWT between different values of MES (*p*=0.06, *p* < 0.05) (Table 2).

**Figure 1:**
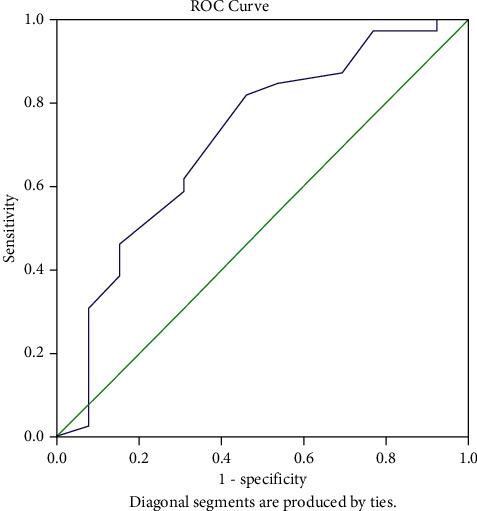
ROC curve analysis of BWT.

Using ROC curve analysis (Figure 1), we calculated BWT for moderately active disease with a cutoff 4.75 mm, with a sensitivity of 82% and specificity of 64% (AUC = 0.713, 95% CI 0.542–0.884).

The overall accuracy of ultrasonography for the disease activity was statistically significant with moderate straight of agreement (*κ* = 0.521, SE (k) = 0.154, *p* < 0.05). Sensitivity of ultrasonography in the assessment of disease activity was 87.8%, while specificity was 83.3%.

Furthermore, logistic regression showed that patients with BWT higher than 4.75 mm have a 5.33-fold chance of being diagnosed with moderately to severely active UC (OR 5.33, 95% CI 1.36–20.84, *p*=0.016, *p* < 0.05).

### 3.6. Correlation of Disease Activity Examined by Ultrasonography and Inflammatory Markers

There was a statistically significant negative correlation of serum albumin with BWT, MES, DAI, DUBLIN, and Geboes index (*r* = -0.375, *p*=0.01, *p* < 0.055) and a positive correlation of CRP with BWT, MES, DAI, DUBLIN, and Geboes index (*r* = 0.323, *p*=0.016, *p* < 0.05). Moreover, we have found a significant correlation of FCP with BWT, MES, DAI, DUBLIN, and Geboes index (*p* < 0.05).

## 4. Discussion

Adequate evaluation of the UC extent and activity, as well as frequent monitoring of patients, is necessary to achieve appropriate disease control. Considering that colonoscopy is still the gold standard for the management of patients with UC, there is a need for the development of reliable, safe, noninvasive methods for the determination of disease severity and localization.

This retrospective study compared the diagnostic accuracy of intestinal US vs. colonoscopy in assessing disease severity and localization in patients with UC. We also investigated the correlation between intestinal US findings and multiple scores which reflect the inflammatory burden of disease in UC as well as inflammatory parameters routinely used in clinical practice [21, 22]. This is the first study of intestinal US validation in relation to the DUBLIN score previously described as a novel score for the evaluation of total inflammatory burden in UC patients.

BWT is the most frequently used US parameter in patients with UC, possibly owing to the fact that colonic mucosal structure is altered due to inflammation. A recent prospective study by Kinoshita et al. showed that intestinal US parameters, BWT predominantly, could be used in the assessment of disease activity, which is in concordance with the results of our investigation [23]. In the systematic review of Smith et al., it has been observed that BWT higher than 4 mm could suggest the presence of UC [24]. Additionally, in the TRUST&UC study, the largest multicenter study on the use of intestinal US in patients with UC, BWT has been recognized as a US parameter for the detection of disease activity [25]. These results are similar to the results of our study, where we have found that the cutoff value of 4.75 mm can suggest moderately active UC. Our measurements of BWT were limited to the sigmoid colon, which often demonstrates a certain degree of hypertrophy of the lamina muscularis propria, a possible explanation for the slightly higher measurement compared to other studies.

In the review of Smith et al., it has been proposed that combining BWT with other US parameters could increase the diagnostic accuracy of intestinal US in UC patients [24]. Our results are in concordance with these findings given that fat wrapping and wall layer stratification were present in the majority of patients with UC in our cohort.

MES is the most commonly used score in endoscopy units worldwide; however, Rowan et al. suggested that the DUBLIN score could improve the evaluation of the disease in UC patients due to its significant correlation with objective inflammatory markers [18]. Our results showed a significant correlation of BWT and disease activity scores, MES, DAI, Geboes index, as well as DUBLIN score, which further emphasize the utility of intestinal US in UC patients.

Evaluation of disease extent is necessary for the patients with UC. Previous findings compared the intestinal US with colonoscopy as well as histopathology reports of both active and inactive diseases [26, 27]. Regarding the assessment of disease extent, our results suggest that moderate concordance exists with intestinal US and colonoscopy. The overall accuracy of intestinal US for disease extension was statistically significant, with a sensitivity of 74.3% and specificity of 80%. These results are similar to the results of Kinoshita et al. as well as Alloca et al. [23,26].

The correlation of US parameters and inflammatory markers has been a field of investigation in multiple studies. Our results suggest a positive correlation of BWT with FCP and CRP as well as a negative correlation with serum albumin, which is similar to previous findings of Rowan et al [18].

### 4.1. Limitations of the Study

This investigation has limitations, such as its retrospective nature and the relatively small sample size. Moreover, measurements of the BWT were limited to sigmoid colon, and only available ultrasonographic data from medical records were included in this study.

## 5. Conclusions

Results of our study suggest that BWT could serve as noninvasive parameter of UC severity as well as UC extent. Taking everything into account, intestinal US could be one of the promising methods for monitoring patients with UC.

## Figures and Tables

**Table 1:** Demographic characteristics of patients with UC.

Variables	Total patients (*n* = 55)
Sex (m/f)	33/22 (60/40)
Age (years)	44.20 ± 17.33
Disease duration average	6.69 ± 6.76
Localization, *n* (%)	
Left-sided	20 (36)
Pancolitis	35 (64)
Disease duration average	6.69 ± 6.76
Laboratory test	
Hb (g/L)^a^	107.96 ± 3.37
WBC (10^9^/L)^b^	9.08 (5.00)
Plt (10^9^/L)^a^	357.89 ± 17.40
Ne (%)^b^	6.28 (4.00)
Ly (%)^b^	1.75 (0.80)
FCP (*μ*g/g)^b^	1337.73 (1066)
D-dimer (mg/L)^b^	1.62 (1.72)
Alb (g/L)^a^	35.96 ± 1.32
CRP (mg/L)^b^	64.81 (86.9)
Pct (ng/L)^b^	0.22 (0.17)
SE (mmol/L)^a^	53.83 ± 6.17
Fe (*μ*mol/L)^b^	6.55 (5.45)
Ferritin (*μ*g/L)^b^	255.09 (335.95)

^a^mean ± SD; ^b^median (IQR). n, number of patients; Hb, hemoglobin; WBC, white blood cell; Plt, platelet; Ne, neutrophils; Ly, lymphocites; Alb, albumin; Fe, serum iron; Cr, creatinine; SE, sedimentation rate; CRP, C-reactive protein; Pct, procalcitonin.

**Table 2:** Mean values of BWT in different groups of Mayo endoscopic score.

	MES 1	MES 2	MES 3	*p* value
BWT (cm) ± SD	2.60 ± 1.78	4.79 ± 2.46	6.15 ± 2.13	0.06

## Data Availability

All data are available from the corresponding author upon request.
